# Management of Renin-Angiotensin-Aldosterone System blockade in patients admitted to hospital with confirmed coronavirus disease (COVID-19) infection (The McGill RAAS-COVID- 19): A structured summary of a study protocol for a randomized controlled trial

**DOI:** 10.1186/s13063-021-05080-4

**Published:** 2021-02-05

**Authors:** Mona Aflaki, Alexandria Flannery, João Pedro Ferreira, Matthew Pellan Cheng, Nadine Kronfli, Ariane Marelli, Faiez Zannad, James Brophy, Jon Afillalo, Thao Huynh, Nadia Giannetti, Amal Bessissow, Justin A. Ezekowitz, Renato D. Lopes, Andrew P. Ambrosy, Morgan Craig, Abhinav Sharma

**Affiliations:** 1 Division of Internal Medicine, McGill University Health Centre, McGill University, Montreal, Quebec Canada; 2 Division of Cardiology, McGill University Health Centre, McGill University, 1001 Decarie Blvd, Montreal, Quebec H4A 3J1 Canada; 3 DREAM-CV Lab, McGill University Health Centre Research Institute, McGill University, Montreal, Quebec Canada; 4 Centre D’Investigation Clinique- Plurithématique Inserm CIC-P 1433, Inserm U1116, CHRU Nancy Hôpitaux de Brabois, F-CRIN INI-CRCT (Cardiovascular and Renal Clinical Trialists), Université de Lorraine, Nancy, France; 5 Division of Infectious Disease, McGill University Health Centre, McGill University, Montreal, Quebec Canada; 6 Division of Cardiology, Jewish General Hospital, McGill University, Montreal, Quebec Canada; 7 Canadian VIGOUR Centre, University of Alberta, Edmonton, Alberta Canada; 8 Duke Clinical Research Institute, Duke University, Durham, North Carolina USA; 9 Department of Cardiology, Kaiser Permanente San Francisco Medical Center, San Francisco, California USA; 10 Division of Research, Kaiser Permanente Northern California, Oakland, California USA; 11 Sainte-Justine University Hospital Research Centre and Department of Mathematics and Statistics, Université de Montréal, Montreal, Quebec Canada

**Keywords:** COVID-19, Randomised controlled trial, Protocol, SARS- CoV2, RAAS inhibitor, ACE2, ACE inhibitor, Mortality, Cardiovascular disease

## Abstract

**Objectives:**

The aim of the RAAS-COVID-19 randomized control trial is to evaluate whether an upfront strategy of temporary discontinuation of renin angiotensin aldosterone system (RAAS) inhibition versus continuation of RAAS inhibition among patients admitted with established COVID-19 infection has an impact on short term clinical and biomarker outcomes. We hypothesize that continuation of RAAS inhibition will be superior to temporary discontinuation with regards to the primary endpoint of a global rank sum score. The global rank sum score has been successfully used in previous cardiovascular clinical trials.

**Trial design:**

This is an open label parallel two arm (1,1 ratio) randomized control superiority trial of approximately 40 COVID-19 patients who are on chronic RAAS inhibitor therapy.

**Participants:**

Adults who are admitted to hospital within the McGill University Health Centre systems (MUHC) including Royal Victoria Hospital (RVH), Montreal General Hospital (MGH) and Jewish General Hospital (JGH) and who are within 96 hours of COVID-19 diagnosis (confirmed via PCR on any biological sample) will be considered for the trial. Of note, the initial protocol to screen and enrol within 48 hours of COVID-19 diagnosis was extended through an amendment, to 96 hours to increase feasibility. Participants have to be 18 years or older and would have to be on RAAS inhibitors for at least a month to be considered eligible for the study. Additionally, RAAS inhibitors should not have been held for more than 48 hours before randomization. A list of inclusion and exclusion criteria can be found in the full protocol document. In order to prevent heart failure exacerbation, patients with reduced ejection fraction were excluded from the trial.

Once a patient is admitted on the ward with a diagnosis of COVID-19, we will confirm with the treating physician if the participant is suitable for the RAAS-COVID trial and meets all the inclusion and exclusion criteria. If the patient is eligible and informed consent has been obtained we will collect data on sex, age, ethnicity, past medical history and list of medications (e.g. other anti-hypertensives or anticoagulants), for further analysis.

**Intervention and comparator:**

All the study participants will be randomized to a strategy of temporarily holding the RAAS inhibitor [intervention] versus continuing the RAAS inhibitor [continued standard of care]. Among participants who are randomized to the intervention arm, alternative guide-line directed anti-hypertensive medication will be provided to the treating physician team (detail in study protocol). In the intervention arm RAAS inhibitor will be withheld for a total of 7 days with the possibility of the withdrawn medication being initiated at any point after day 7 or on the day of discharge. The recommendation for re-initiating the withdrawn medication will be made to the treating physician. The re-initiation of these therapies are according to standard convention and follow-up as per Canadian guidelines. Additionally, the date of restarting the withdrawn medication or whether the medication was re-prescribed on discharge or not, will be collected. This will be used to conduct a sensitivity analysis. Furthermore, biomarkers such as troponin, c-reactive protein (CRP) and lymphocyte count will be assessed during the same time period. Samples will be collected on randomization, day 4 and day 7.

**Main outcomes:**

**Primary endpoint:**

In this study the primary end point is a global rank score calculated for all participants, regardless of treatment assignment ( score from 0 to 7). Please refer to table 4 in the full protocol. In the context of the current trial, it is estimated that death is the most meaningful endpoint, and therefore has the highest score ( score of 7). This is followed by admission to ICU, the need for mechanical ventilation etc. The lowest scores ( score of 1) are assigned to biomarker changes (e.g. change in troponin, change in CRP). This strategy has been used successfully in cardiovascular disease trials and therefore is applicable to the current trial.

The primary endpoint for the present trial is assessed from baseline to day 7 (or discharge). Participants are ranked across the clinical and biomarker domains. Lower values indicate better health (or stability). Participants who died during the 7th day of the study will be ranked based on all events occurring before their death and also including the fatal event in the score. Next, participants who did not die but were transferred to ICU for invasive ventilation will be ranked based on all the events occurring before the ICU entry and also including the ICU admission in the score. Those participants who did not die were not transferred to ICU for invasive ventilation, will be ranked based on the subsequent outcomes. The mean rank score will then be compared between groups. In this scheme, a lower mean rank score indicates greater overall stability for participants.

Secondary endpoints :

The key secondary endpoints are the individual components of the primary components and include the following: death, transfer to ICU primarily for invasive ventilation, transfer to ICU for other indication, non-fatal MACE ( any of following, MI, stroke, acute HF, new onset Afib), length of stay > 4 days, development of acute kidney injury ( > 40% decline in eGFR or doubling of serum creatinine), urgent intravenous treatment for high blood pressure, 30% increase in baseline high sensitivity troponin, 30% increase in baseline BNP, increase in CRP to > 30% in 48 hours and lymphocyte count drop> 30%. We will also look at the World Health Organization (WHO) ordinal scale for clinical improvement (in COVID-19) in our data. In this scale death will be assigned the highest score of 8. Patients with no limitation of activity will be assigned a score of 1 which indicates overall more stability (3).

Additionally, we will evaluate the potential effects of discontinuing RAAS inhibition on alternative schedules (longer/shorter than 7 days, intermittent discontinuation) using a mechanistic mathematical model of COVID-19 immunopathology calibrated to data collected from our patient cohort. In particular, we will assess the impact of alternative schedules on primary and secondary endpoints including increases to baseline CRP and lymphocyte counts.

**Randomization:**

Participants will be randomized in a 1:1 ratio. Randomization will be performed within an electronic database system at the time of enrolment using a random number generator, an approach that has been successfully used in other clinical trials. Neither participant, study team, or treating team will be blinded to the intervention arm.

**Blinding:**

This is an open label study with no blinding.

**Numbers to be randomised (sample size):**

The approximate number of participants required for this trial is 40 patients (randomized 1:1 to continuation versus discontinuation of RAAS inhibitors). This number was calculated based on previous rates of outcomes for COVID-19 in the literature (e.g. death, ICU transfer) and statistical power calculations.

**Trial Status:**

Protocol number: MP-37-2021-6641, Version 4: 01-10-2020. Trial start date September 1^st^ 2020 and currently enrolling participants. Estimated end date for recruitment of participants : July 2021. Estimated end date for study completion: September 1^st^ 2021.

**Trial registration:**

Trial registration: ClincalTrials.gov: NCT04508985, date of registration: August 11^th^ , 2020

**Full protocol:**

The full protocol is attached as an additional file, accessible from the Trials website (Additional file 1). In the interest in expediting dissemination of this material, the familiar formatting has been eliminated; this Letter serves as a summary of the key elements of the full protocol.

**Supplementary Information:**

The online version contains supplementary material available at 10.1186/s13063-021-05080-4.

## Supplementary Information

**Additional file 1.**

## Data Availability

De-identified participant data is stored on a secure password protected red-cap data base. Access to the data base will be controlled by the principal investigator. Access criteria to the final trial dataset will be provided upon written request to the corresponding author/principal investigator on publication.

