# Supplementary material for: Management of Renin-Angiotensin-Aldosterone System blockade in patients admitted to hospital with confirmed coronavirus disease (COVID-19) infection (The McGill RAAS-COVID- 19): A structured summary of a study protocol for a randomized controlled trial
Source: Trials. 2021 Feb 5;22:115. doi: 10.1186/s13063-021-05080-4 (PMC7862851; doi:10.1186/s13063-021-05080-4)
Supplement: Supplementary file 1 — Additional file 1. [file 13063_2021_5080_MOESM1_ESM.pdf]

Management of **R**enin-**A**ngiotensin-**Al**dosterone **S**ystem blockade in patients admitted in hospital with confirmed coronavirus disease (**COVID-19**) infection: **The McGill RAAS-COVID-19 randomized controlled trial**

Abhinav Sharma, MD, PhD[1]; Nadine Kronfli MD, MPH[2]; Ariane Marelli MD, MPH[1]; James Brophy MD, PhD[1]; Jon Afillalo MD, MSc;[4] Thao Huynh MD;[1] Nadia Giannetti MD[1], Amal Bessissow MD[3], Matthew Cheng[2]

1 Division of Cardiology, McGill University Health Centre, McGill University, Montreal, Canada
2. Division of Infectious Disease, McGill University Health Centre, McGill University, Montreal, Canada
3. Division of Internal Medicine, McGill University Health Centre, McGill University, Montreal, Canada
4. Division of Cardiology, Jewish General Hospital, McGill University, Montreal, Canada

Protocol number: MP-37-2021-6614

Version 4: 01-10-2020

Table of Content

1. EXECUTIVE SUMMARY 3
2. FUNDING 4
3. INTRODUCTION 4
BACKGROUND AND SIGNIFICANCE 4
4. OBJECTIVES AND HYPOTHESES 5
5. JUSTIFICATION OF EQUIPOISE 5
6. EXTERNAL SCIENTIFIC REVIEW 6
7. STUDY DESIGN 6
8. STUDY POPULATION AND ELIGIBILITY CRITERIA 6
INCLUSION CRITERIA 6
EXCLUSION CRITERIA 6
9. STUDY INTERVENTIONS 6
10. RANDOMIZATION AND BLINDING 7
11. RECRUITMENT AND SCREENING PROCEDURES 7
RECRUITMENT AND ENROLLMENT 7
PRIMARY ENDPOINT 8
SAMPLE SIZE CALCULATION 9
SECONDARY ENDPOINTS 9
12. STATISTICAL ANALYSIS PLAN 9
13. BASELINE EVALUATION AND RANDOMIZATION 10
RANDOMIZATION AND DETERMINATION OF ENDPOINTS 10
BIOMARKERS 10
14. INSTITUTIONAL REVIEW BOARDS 11
15. INFORMED CONSENT 11
16. WITHDRAWAL OR DISCONTINUATION OF PARTICIPANTS 11
POTENTIAL RISKS AND MITIGATION STRATEGY 11
17. DATA MANAGEMENT PROCEDURES 11
OVERVIEW OF DATA MANAGEMENT 11
18. DATA QUALITY CONTROL 11
19. DATA SHARING 12
20. DATA SECURITY 12
21. REGULATORY ISSUES 12
ETHICS AND GOOD CLINICAL PRACTICE 12
22. BLOOD SPECIMEN HANDLING 12
BLOOD COLLECTION AND HANDLING IN THE HOSPITALS 12
23. FUNDING 12
24. IMPLICATIONS 13
25. LITERATURE CITED 13
26. APPENDIX 15
APPENDIX: CTN CIHR CANADIAN HIV TRIALS NETWORK COMMENTS 15

APPENDIX TABLE 1:..........18
APPENDIX TABLE 2:..........19
APPENDIX TABLE 3: CASE REPORT FORM..........19

1. Executive Summary

Title	Management of **R**enin-**A**ngiotensin-**A**ldosterone **S**ystem blockade in patients admitted in hospital with confirmed coronavirus disease (**COVID-19**) infection: **The McGill RAAS-COVID-19 randomized controlled trial**
Indication	Patients admitted with SARS-CoV-2 (COVID-19) infection
Location	McGill University Health Center (MUHC) at the Royal Victoria Hospital (RVH) and Montreal General Hospital (MGH). CIUSSS du Centre-Ouest-de-L'Ile-de-Montréal at the Jewish General Hospital (JGH).
Lay Summary	Coronavirus disease (COVID-19) related pneumonia significantly impact patients with underlying cardiovascular (CV) conditions. Animal studies suggest that drugs commonly used to treated CV diseases may increase the ability of COVID-19 to infect cells. The RAAS-COVID-19 trial aims to assess whether temporarily holding these CV drugs on admission, versus continuing them, in patients admitted with COVID-19 can impact outcomes.
Brief Rationale	Among patients with underlying CV diseases (namely hypertension, prior myocardial infarction [MI], and heart failure), renin-angiotensin-aldosterone (RAAS) system inhibitors are among the most frequently prescribed medications. Recent pre-clinical data suggests that ACEi could act as a risk factor for fatal SARS-CoV-2 infection.[1] As previously shown for SARS-CoV[2], viral cell entry for SARS-CoV-2 utilizes angiotensin-converting enzyme 2 (ACE2).[1] Similarly, recently emerging data also suggests that SARS-CoV-2 also engages ACE2 as an entry receptor into cells.[1] In pre-clinical and clinical studies, ACEi and ARB significantly upregulates[3,4] These observations bring up the critical question of whether RAAS inhibition may increase the risk of deleterious outcome of COVID-19 through up-regulation of ACE2 and increase of viral load, potentially suggesting that RAAS inhibition should be held.[5] However, holding RAAS inhibitors may induce clinical CV deterioration. All consensus guidelines urge that more evidence is needed in order to provide robust clinical practice recommendations for RAAS use in COVID-19 infected patients.[6,7] The RAAS-COVID-19 trial will provide guidance on the use of RAAS inhibitors in patients with COVID-19 infection.
Primary Aim	The RAAS-COVID-19 RCT will evaluate whether an upfront strategy of temporary discontinuation of RAAS inhibition compared to continuation of RAAS inhibition among patients admitted with established COVID-19 infection and on chronic RAAS inhibition therapy impacts short term clinical outcomes and biomarkers.
Patient Population	Patients admitted in hospital with COVID-19 infection who are being treated with RAAS inhibitors.
Study Design	Open label, randomized, study of 40 adults. The following groups of participants will be considered: i) within 96 hours of diagnosis of COVID-19; ii) who have received a diagnosis of COVID-19 from another facility and are within 96 hours of transfer to a study recruitment site (RVH, MGH, JGH). Participants will be randomized 1:1 to an upfront temporary discontinuation)

	of RAAS inhibition for the duration of the hospitalization (and to consider re-initiate on discharge) versus a strategy continuation of RAAS inhibition.
Interventions	Withdrawal of RAAS inhibitors for the duration of hospitalization
Primary Endpoint	The primary end point is a global rank score which is applied to all participants, regardless of treatment assignment. The primary endpoint will be assessed from baseline to day 7 (or discharge). The global rank sum score will be developed based on a tiered endpoints of outcome events which includes clinical and biomarker endpoints. Participants will receive a weighted score depending on the first event experienced between baseline to day 7 (or discharge). The global rank sum score will then be averaged and compared between treatment arms.
Component and weight of primary endpoints	(see table below)

Component and weight of primary endpoints:

Item (from Randomization to day 7/discharge for primary outcome)	Points
Death	7
Transfer to ICU for Invasive ventilation	6
Transfer to ICU for other indication	5
Non-fatal MACE (Any of the following - MI, Stroke, Acute HF, new onset Afib)	4
Length of stay > 4 days	3
Development of acute kidney injury (>40% decline in eGFR or doubling of serum Cr)	2
Urgent intravenous treatment for high blood pressure/hypertensive crisis	2
>30% increase in baseline high sensitivity troponin	1
>30% increase in baseline BNP	1
Increase in baseline CRP to 48 hours >30%	1
Lymphocyte count drop >30%	1
ICU: intensive care unit; MACE major adverse cardiac event; MI myocardial infarction; Afib atrial fibrillation; eGFR estimated glomerular filtration rate calculated by the Modification of Diet in Renal Disease Study (MDRD) equation; BNP brain natriuretic peptide; CRP c-reactive protein	

2. Funding

This study is funded by the McGill Interdisciplinary Initiative in Infection and Immunity (MI4) and the McGill University Health Centre Division of Cardiology. The funders will not play any role in the conduct of the study.

3. Introduction

Background and Significance

Increasing global experience with the coronavirus (COVID-19) pandemic suggests that older patients with underlying CV disease are at 3-4 fold higher risk of adverse outcomes including intensive care admission, need for invasive mechanical support, need for inotropic support, and mortality.[8–10] Given the high degree of morbidity and mortality in this population, a critical public health issue will be the management of CV disease among patients with established COVID-19 infection.

Among patients with underlying CV diseases (namely hypertension, prior myocardial infarction [MI], and HF), renin-angiotensin-aldosterone (RAAS) system inhibitors are among the most frequently prescribed medications. Recent pre-clinical data suggests that angiotensin converting enzyme inhibitors (ACEi) and angiotensin receptor blockers (ARBs) could act as a risk factor for fatal SARS-CoV-2 infection.[1] As previously shown for SARS-CoV[2], viral cell entry for SARS-CoV-2 utilizes angiotensin-converting enzyme 2 (ACE2) receptors.[1] Similarly, recently emerging data also suggests that SARS-CoV-2 also engages ACE2 as an entry receptor into cells.[1] In pre-clinical and clinical studies, ACEi and ARBs significantly upregulates[3,4] ACE2 receptors. ACE2 is a homolog of ACE. ACE2 negatively regulates the renin angiotensin system by converting Angiotensin II to vasodilatory Angiotensin 1-7, thereby diminishing and opposing the vasoconstrictor effect of angiotensin II. Interactions between ACE2, ACE, angiotensin II and other renin angiotensin aldosterone system (RAAS) system are complex, and tissue expression of ACE2 differs depending on the underlying organ and patient host. Given the complexity of the interaction of the RAAS system and ACE2, the kinetics of addition or withdrawal of RAAS inhibitors and the impact on subsequent ACE2 levels in human tissue remain unclear.[11–13]

Currently, there are no experimental or clinical data demonstrating beneficial or adverse outcomes with the use of ACE inhibitors, ARBs or other RAAS antagonists in COVID-19 or among COVID-19 patients with a history of cardiovascular disease. Until further evidence is established, all consensus guidelines recommend the continuation of RAAS antagonists for patients who require them for heart failure, hypertension, or ischemic heart disease.[14,15]

Despite international recommendations to continue RAAS antagonists, determining whether RAAS inhibition may increase the risk of deleterious outcomes among COVID-19 patients should be determined. If so, management of patients with COVID-19 could change to suggest that RAAS inhibition should be held during acute COVID-19 infection.[5] However, withholding RAAS inhibitors may potentially induce clinical CV deterioration. All consensus guidelines urge that more evidence is needed in order to provide robust clinical practice recommendations for RAAS use in COVID-19 infected patients.[6,7] A recently published observational study suggests that patients on ACEi/ARB have improved outcomes; however, the inherent in observational studies prevents ascertainment of causation.[16] The RAAS-COVID-19 trial will provide guidance on the use of RAAS inhibitors in patients with COVID-19 infection.

4. Objectives and Hypotheses

The RAAS-COVID-19 RCT will evaluate whether an upfront strategy of temporary discontinuation of RAAS inhibition versus continuation of RAAS inhibition among patients admitted with established COVID-19 infection impacts on short term clinical and biomarker outcomes. We hypothesize that continuation of RAAS inhibition will be superior to temporary discontinuation with regards to the primary endpoint of a global rank sum score (see endpoints below).

5. Justification of Equipoise

There is significant uncertainty with regards to the role of RAAS inhibition in patients with COVID-19.[17] Indeed there are both trials currently being designed that are initiating ARBs in patients with COVID-19 (https://clinicaltrials.gov/ct2/show/NCT04312009) and others that are withdrawing RAAS inhibitors in patients with COVID-19 (https://clinicaltrials.gov/ct2/show/NCT04329195?cond=covid&draw=8). Given the controversy in the literature regarding RAAS inhibition in the context of an active COVID-19 infection, the RAAS-COVID-19 has sufficient equipoise to be conducted.

6. External Scientific Review

The current protocol has undergone external scientific review from the CTN-CIHR HIV Clinical trials network in elicit external feedback. We received significant supportive comments and we have modified our protocol to ensure scientific validity. Please see the attached files for comments from the external reviewers and the appendix for response and our modifications to the reviewer comments. We feel that our protocol is now significantly strengthened after this external scientific review.

7. Study Design

This is an open labeled two arm randomized controlled trial.

8. Study Population and Eligibility Criteria

Inclusion Criteria

- Age ≥ 18 years old.
- Hospitalization with a Covid-19 infection
- Chronically treated with RAAS blockers (ACE inhibitors or ARBs on the last prescription prior to admission with a treatment duration ≥ 1 month; [see appendix table 1 for definition of RAAS inhibitors])
- Diagnosis of COVID-19 confirmed by the presence of SARS-CoV-2 on any biological sample
- Participants are within 96 hours of diagnosis or admission for COVID-19 or have received a diagnosis of COVID-19 from another facility and are within 96 hours of transfer to a study recruitment site

Exclusion Criteria

- Shock requiring vasoactive agents.
- Requiring invasive mechanical ventilation.
- History of malignant hypertension
- Use of five or more antihypertensive drugs.
- History of heart failure with reduced ejection fraction
- History of hospitalization for acute heart failure in past 3 months
- History of hospitalization for hemorrhagic stroke in the past 3 months.
- History of CKD with an eGFR <45 ml/min/1.73m2
- History of COPD GOLD III/IV
- History of end-stage dementia
- History of active liver cirrhosis
- RAAS blockers therapy previously stopped > 48h.
- Anticipated discharge in less than 24 hours.
- History of current active cancer receiving chemotherapy
- Inability to obtain informed consent.

9. Study Interventions

All the study participants all be randomized to a strategy of temporarily holding the RAAS inhibitor [intervention] versus continuing the RAAS inhibitor [continued standard of care]. Among participants who will be randomized to the intervention arm, possible guideline directed alternative to anti-hypertensive medication alternatives will be provided to the treating physician team (see appendix table 2). The recommendation to restart the RAAS inhibitor upon anticipated discharge will also be made. All subsequent treatment recommendations will be left up to the treating team. The withdrawn medication can be initiated at any point after day 7 or on the day of discharge if the participant is being discharged prior to day 7. This will have to be

prescribed at the clinical discretion of the treating team. The study team will be communicating with the treating team daily to indicate to the treating team physician that the medication has been withdrawn and to consider restarting the medication after day 7 or day of discharge. The re-initiation of these therapies will follow standard convention and follow-up as per Canadian guidelines. In addition, we will collect the date of restarting the withdrawn medication on the CRF and/or if the medication was prescribed on discharge. We will conduct a sensitivity analysis whereby we will adjust the results based on date of restarting medication and whether the withdrawn medication was prescribed on discharge.

10. Randomization and Blinding

Participants will be randomized in a 1:1 ratio. Randomization will be performed within an electronic database system at the time of enrollment using a random number generator, an approach we have used successfully in other clinical trials. Neither participant, study team, or treating team will be blinded.

11. Recruitment and Screening Procedures

Recruitment and Enrollment

We will include adult participants (approximately 40) who are admitted in hospital within the MUHC systems (Royal Victoria Hospital [RVH], Montreal General hospital [MGH]) and the CIUSSS de l'Ouest-de-l'Ile-de-Montréal (Jewish General Hospital [JGH]). Our team will initially approach the treating team physician to identify if there are any possible participants who would meet the eligibility for our study. The patient's chart will then be screened to determine if they meet full inclusion and exclusion criteria. We will confirm with the treating team if the participant is suitable for recruitment in the RAAS-COVID-19 trial. Following randomization, the study team will inform the treating team which study arm the participant has been assigned. If the participant has be randomized to the arm whereby the RAAS inhibitor is withdrawn, the RAAS-COVID-19 site PI will provide recommendation (see appendix) on alternative guideline recommended drug treatments. The study team site PI will be available to discuss provide any expertise regarding the participants clinical condition following withdrawal of the RAAS inhibitors. An information and consent form (ICF) will initially be given to the participant by the clinical nurse, due to the limited access to the COVID unit. The participant will then be contacted by the study nurse or coordinator where the study will be explained. Verbal informed consent will be obtained in the presence of the witness. The informed consent will be signed by both the study nurse or coordinator and the witness. The participant will then be randomized in the study and a copy of the ICF will be placed in the participants chart.

Coordination between studies

Given the number of studies (both interventional and observational) that will be simultaneously on-going, the study teams will coordinate our approach to ensure a minimal number of contacts with the participants. The treating team recognizes the multiple on-going trials. If a patient is already enrolled in a randomized clinical trial, we will not approach this participant unless referred by another study PI. If a participant enrolled in the RAAS-COVID-19 trial is being considered for enrollment in another clinical trial, we will allow for co-enrollment in that trial assuming the participant meets all inclusion and exclusion criteria of that trial. We are collecting whether participants are being enrolled in additional trials in our CRF. The study team for RAAS-COVID-19 will make the protocol available for review by site PIs of other trials.

ICU: intensive care unit; MACE major adverse cardiac event; MI myocardial infarction; Afib atrial fibrillation; eGFR estimated glomerular filtration rate calculated by the Modification of Diet in Renal Disease Study (MDRD) equation; BNP brain natriuretic peptide; CRP c-reactive protein

Item (from Randomization to day 4 for primary outcome)	Points
Death	7
Transfer to ICU for invasive ventilation	6
Transfer to ICU for other indication	5
Non-fatal MACE (Any of the following - MI, Stroke, Acute HF, new onset Afib)	4
Length of stay > 4 days	3
Development of acute kidney injury (>40% decline in eGFR or doubling of serum Cr)	2
Urgent intravenous treatment for high blood pressure/hypertensive crisis	2
>30% increase in baseline high sensitivity troponin	1
>30% increase in baseline BNP	1
Increase in baseline CRP to 48 hours >30%	1
Lymphocyte count drop >30%	1

Primary Endpoint

There is clearly an unmet need to define better "intermediate" end points—that is, end points that can predict the effect (or lack thereof) of an intervention to capture both early clinical events and important pathophysiologic phenomena that might meaningfully reflect "downstream" events.[18–20] These endpoints would then provide greater context for a larger trial which would require a larger sample size and would take more time to provide results. Such an intermediate end point should be quantitative, be reproducible, and have sufficient power to allow for reasonable sample size in pilot/early studies. Incorporating clinical and pathophysiologic endpoints (such as biomarkers) into end points in clinical trials requires a framework for combining both clinical events and continuous data into a unified metric. This is in contrast to most end points in phase III clinical trials which typically use a "time-to-event" analyses. Given that pilot studies are, by definition, underpowered to conclusively demonstrate significant differences on clinical end points, alternative approaches are necessary. The global rank sum score is one such approach is to examine the results across multiple clinical end points, recognizing that none are likely to reach nominal statistical significance. Using a global rank sum score strategy enables basing an endpoint decision on the totality of observed trends across multiple clinical and biomarker domains.[20] In this method, all participants participating in a clinical trial (regardless of treatment assignment) are ranked on the basis of a prespecified hierarchical ranking of outcomes. Such components could include both "events" (such as death or length of hospitalization) and quantitative assessments such as biomarker measurements which has established prognostic value (e.g. natriuretic peptides, lymphocytes count etc). Each individual endpoint is assigned a score based on estimation of the value of that score. In the context of the current trial, we estimate that death is the most meaningful endpoint, therefore has the highest score. This is followed by admission to ICU, the need for mechanical ventilation etc. The lowest scores are assigned to biomarker changes. Each participant accrues a score. The totality of the median scores across each randomized arms will be compared using a wilcox rank sum test. This strategy has been used in cardiovascular disease trials and can be readily used in the present scenario.[20,21]

The primary end point is a global rank score in which all participants, regardless of treatment assignment. The global rank sum score is an alternative endpoint to traditional time to outcome analysis or binary endpoint ascertainment.[18] The global rank sum score enables higher weighting to be given to endpoints perceived to be of greatest importance.[22] The primary

endpoint for the present trial will be assessed from baseline to day 7 (or discharge). Participants are ranked across the clinical and biomarker domains (**see table 1**)

Lower values indicate better health (or stability). Participants who died during the 7th day of the study will be ranked based on all events occurring before their death and also including the fatal event in the score. Next, participants who did not die but were transferred to ICU for invasive ventilation will be ranked based on all the events occurring before the ICU entry and also including the ICU admission in the score. Those participants who neither died nor were transferred to ICU for invasive ventilation will be ranked based on the subsequent outcomes (and so on for all of the outcomes). The mean rank score will then be compared between groups. In this scheme, a lower mean rank score indicates greater overall stability for participants.

Sample size calculation
Given prior rates of outcomes from COVID-19 literature[10,23–25] we estimate the following among participants admitted with COVID-19: 27% will require an ICU admission, 25% will require mechanical ventilation, and 28% will die. Based on these assumptions, we estimate a mean of 16 points in the control group and a reduction to 12 points in the experimental group with a standard deviation of 5 points. To meet these assumptions 40 participants are required to have a 80% chance of detecting (at the 5% level), a decreases in the primary outcome from 16 to 12 points (as above described).

Secondary endpoints
The key secondary endpoints are the individual components of the primary components and include the following:

- Death
- Transfer to ICU primarily for Invasive ventilation
- Transfer to ICU for other indication
- Non-fatal MACE (Any of the following - MI, Stroke, Acute HF, new onset AfIb)
- Length of stay > 4 days
- Development of acute kidney injury (>40% decline in eGFR or doubling of serum Cr)
- Urgent intravenous treatment for high blood pressure/hypertensive crisis
- >30% increase in baseline high sensitivity troponin
- >30% increase in baseline BNP
- Increase in baseline CRP to 48 hours >30%
- Lymphocyte count drop >30%

Exploratory endpoints:
We will assess each individual component of the endpoint up to day 7 or day of discharge.

12. Statistical analysis plan

All analyses will be conducted using the intention-to-treat principle and will include all randomized participants. Analysis of the global rank end point was based on the Wilcoxon test statistic. Medians, 25th and 75th percentiles will be presented for continuous variables; the number and percentage of participants in each category will be presented for categorical variables. For all endpoints a p-value ≤0.05 will be considered statistically significant. Appropriate statistical models will be used to examine the effect of the withdrawal intervention on both the primary, secondary, and exploratory outcomes. For the global rank-based endpoints, a non-parametric testing strategy will be employed. For continuous endpoint variables, conventional general linear models will be used. For endpoints where the response is dichotomous (binary), the logistic regression model will be used. For the primary analysis, we

will conduct several sensitivity analyses: 1) The primary analysis will be adjusted by a time-varying covariate on whether the RAAS inhibitor was restarted and if was prescribed on discharge; 2) we will adjust for whether an individual was co-enrolled in another trials; 3) we will remove individuals who are co-enrolled in additional trials to see if the results vary from the initial analysis.

13. Baseline Evaluation and Randomization

Randomization and determination of endpoints

We will be following the follow schedule for our study

Timeline of the study

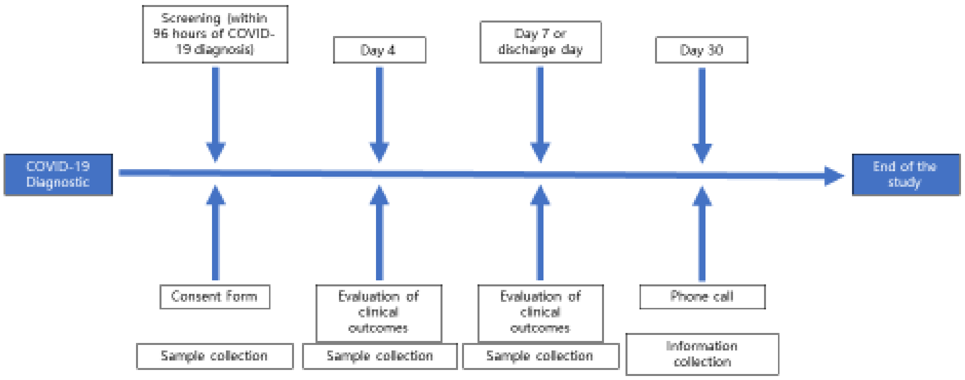

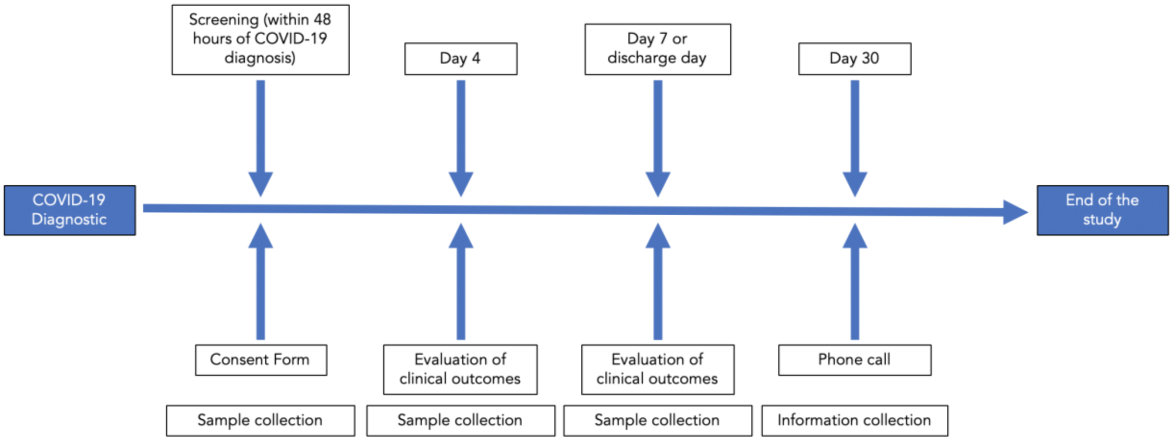

Biomarkers

We will be assessing troponin, brain natriuretic peptide, c-reactive protein, and lymphocyte count. These biomarkers have been previously assessed to have prognostic value within

COVID-19 infections.[9,10,26] If participants are discharged prior to day 7, the day of discharge laboratory investigation will count towards the final endpoint.

14. Institutional Review Boards

The study site will submit the study protocol, informed consent form, and other relevant study documents to their Institutional Review Board (IRB) for approval.

15. Informed Consent

All patients will have the purpose of the study, the study interventions and evaluations, and the potential risks and benefits of participation explained to them and their questions will be answered. An informed consent form (ICF) will be provided to the participant so that trial information and risk /benefits can be reviewed. As an alternative to written consent, verbal consent will be sought from the patient describing in detail the study procedures and risks and answer any questions they might have. This will be witnessed by another impartial witness. If verbal consent was given, the care team will document this on the ICF which can be given to the patient at the time of discharge. Given the nature of COVID-19 infection, the study team will obtain verbal consent with the participant over the phone with a three-way phone call: the participant, study team, and witness. Both the witness and study team member obtaining the verbal consent will sign the ICF. A copy of the ICF will be placed into the chart. In the case where the participant requires a legally authorized representative, this will be indicated in the ICF and the same three-way phone communication will occur with the legally authorized representative prior to any study-related assessments or procedures are conducted. As visitors are not allowed in the hospital environment, obtaining a signature from the legally authorized representative would be prohibitive. Participants will be given the opportunity to think about it prior to agreeing to participate and may request that other individuals (e.g. such as next of kin) be aware of the study risks as well. They may withdraw consent at any time throughout the course of the study. Prior to involvement in any study-related activities, consent must be obtained verbally for each participant using the current REB approved informed consent form.

16. Withdrawal or Discontinuation of Participants

Participants may withdraw at any time during the study without prejudice. In addition, a patient may be withdrawn by the investigator is the participant violates the study plan or for administrative reasons. The investigator or designee will document in the case report form (CRF) when a participant has been discontinued or withdrawn from study. When a participant discontinues or is withdrawn before study completion, all applicable activities scheduled for the final study visit should be performed at the time of discontinuation.
Potential Risks and Mitigation Strategy

17. Data Management Procedures

Overview of Data Management
The principle investigators will have primary responsibility for data management, including the development of data collection systems, data monitoring processes, and data storage and back-up. The principle investigators team will develop the CRF modules necessary for this study. A CRF will be developed on a secure REDCAP database which will be stored on an MUHC-RI research computer belonging to the principle investigator. The data collected on the CRF is identified in the appendix table 3.

18. Data Quality Control

A two-step approach to data quality control will be implemented.

1. <u>Training</u>: Prior to the start of enrollment, the Principle Investigators and lab teams will have a virtual introductory meeting to identify the participant workflow through the study.

2. Monitoring: As no drugs are administered per protocol, the principle investigators will conduct data review as the data becomes available. Having frequent phone and e-mail communication with P.I. and study personnel will allow the monitor to assess study status, to provide in-service training, and to address questions from site investigators and coordinators.

19. DATA SHARING

We intend to make the data available to the research community, Researchers from the MUHC, international centers, other universities. Only researchers with approved projects and with a data-sharing agreement will be granted access to the data. We will not provide researchers participants' names or any other information that could directly identify them.

20. Data Security

Access to databases will be controlled centrally by the Principle Investigators through user passwords linked to appropriate privileges. Baseline clinical data will be collected into the CRF and stored on a password protected red-cap database that will be developed and stored on an MUHC-RI research computer. This database will be password protected research computer that can only be accessed by the Principle Investigators. This protects the data from unauthorized changes and inadvertent loss or damage. Database through controlled physical access. All disk drives will be protected using virus-scanning software.

21. Regulatory issues

Ethics and Good Clinical Practice
This study must be carried out in compliance with the protocol. These procedures are designed to ensure adherence to Good Clinical Practice, as described in the following documents:

1. ICH Harmonized Tripartite Guidelines for Good Clinical Practice (ICH E6) 1996.
2. US 21 Code of Federal Regulations Title 45 Part 46 Protection of Human Subjects.dealing with clinical studies (including parts 50 and 56 concerning informed consent and IRB regulations).

Participating investigators agree to follow adhere to the instructions and procedures described in the protocol and, thereby, to adhere to the principles of Good Clinical Practice to which the protocol that it conforms to. The informed consent form(s) must be submitted by the investigator for IRB approval. Any changes to the model consent form suggested by the Investigator must be agreed to by IRB.

22. Blood Specimen Handling

Blood Collection and Handling in the Hospitals
For the purpose of this study, we will coordinate the handling of blood specimens with the research laboratory of each sites (RVH, MGH, JGH). Blood specimen acquisition will be timed so that samples can be drawn alongside clinical care. These tubes will be labelled with the patient’s name and unit number. The tubes will be filled by the nurse or phlebotomist using standard clinical care protocols for obtaining research COVID-19 samples.

23. Funding

This study is sponsored by the McGill Interdisciplinary Initiative in Infection and Immunity (MI4) and the McGill University Health Centre Division of Cardiology.

24. Implications

There is significant uncertainty regarding the use of RAAS inhibitors in people with active COVID-19 infections. The RAAS-COVID-19 trial will provide pivotal clinical information which will inform larger and more adequately powered randomized controlled trials. Furthermore, this data will enable a greater understanding of the dynamics of short-term clinical outcomes and biomarker trends associated with withdrawl versus continuation of RAAS inhibitors in patients admitted with COVID-19 infection.

25. Literature Cited

1. Hoffmann, M. *et al.* The novel coronavirus 2019 (2019-nCoV) uses the SARS-coronavirus receptor ACE2 and the cellular protease TMPRSS2 for entry into target cells. *bioRxiv* 2020.01.31.929042 (2020). doi:10.1101/2020.01.31.929042
2. Li, W. *et al.* Angiotensin-converting enzyme 2 is a functional receptor for the SARS coronavirus. *Nature* **426**, 450–454 (2003).
3. Ferrario, C. M. *et al.* Effect of angiotensin-converting enzyme inhibition and angiotensin II receptor blockers on cardiac angiotensin-converting enzyme 2. *Circulation* **111**, 2605–2610 (2005).
4. Agata, J. *et al.* Olmesartan is an angiotensin II receptor blocker with an inhibitory effect on angiotensin-converting enzyme. *Hypertens. Res.* **29**, 865–874 (2006).
5. Kuster, G. M., Osswald, S., Haaf, P. & Widmer, A. F. SARS-CoV2 : should inhibitors of the renin – angiotensin system be withdrawn in patients with COVID-19 ? 1–3 (2020). doi:10.1093/eurheartj/ehaa235
6. Sharma, A. *et al.* Heart failure event definitions in drug trials in patients with type 2 diabetes. *The Lancet Diabetes and Endocrinology* **4**, 294–296 (2016).
7. Marquis-Gravel, G. *et al.* Technology-Enabled Clinical Trials: Transforming Medical Evidence Generation. *Circulation* **140**, 1426–1436 (2019).
8. Wu, Z. & McGoogan, J. M. Characteristics of and Important Lessons From the Coronavirus Disease 2019 (COVID-19) Outbreak in China: Summary of a Report of 72 314 Cases From the Chinese Center for Disease Control and Prevention. *Jama* **2019**, 3–6 (2020).
9. Guan, W.-J. *et al.* Clinical Characteristics of Coronavirus Disease 2019 in China. *N. Engl. J. Med.* 1–13 (2020). doi:10.1056/NEJMoa2002032
10. Arentz, M. *et al.* Characteristics and Outcomes of 21 Critically Ill Patients With COVID-19 in Washington State. *Jama* **4720**, 2019–2021 (2020).
11. Ye, M. *et al.* Glomerular localization and expression of angiotensin-converting enzyme 2 and angiotensin-converting enzyme: Implications for albuminuria in diabetes. *J. Am. Soc. Nephrol.* **17**, 3067–3075 (2006).
12. Soler, M. J. *et al.* Localization of ACE2 in the renal vasculature: Amplification by angiotensin II type 1 receptor blockade using telmisartan. *Am. J. Physiol. - Ren. Physiol.* **296**, (2009).
13. Vuille-Dit-Bille, R. N. *et al.* Human intestine luminal ACE2 and amino acid transporter expression increased by ACE-inhibitors. *Amino Acids* **47**, 693–705 (2015).
14. Vaduganathan, M. *et al.* Renin-Angiotensin-Aldosterone System Inhibitors in Patients with Covid-19. *N. Engl. J. Med.* NEJMsr2005760 (2020). doi:10.1056/NEJMsr2005760
15. Bavishi, C., Maddox, T. M. & Messerli, F. H. Coronavirus Disease 2019 (COVID-19) Infection and Renin Angiotensin System Blockers. *JAMA Cardiol.* **19**, 1965–1974 (2020).
16. Zhang, P. *et al.* Association of Inpatient Use of Angiotensin Converting Enzyme Inhibitors and Angiotensin II Receptor Blockers with Mortality Among Patients With Hypertension Hospitalized With COVID-19. *Circ. Res.* CIRCRESAHA.120.317134 (2020). doi:10.1161/CIRCRESAHA.120.317134
17. Guo, J., Huang, Z., Lin, L. & Lv, J. Coronavirus Disease 2019 (COVID-19) and Cardiovascular Disease: A Viewpoint on the Potential Influence of Angiotensin-

Converting Enzyme Inhibitors/Angiotensin Receptor Blockers on Onset and Severity of Severe Acute Respiratory Syndrome Coronavirus 2 Infec. *J. Am. Heart Assoc.* **9**, 1–5 (2020).
18. Brown, P. M. & Ezekowitz, J. A. Composite End Points in Clinical Trials of Heart Failure Therapy: How Do We Measure the Effect Size? *Circ. Hear. Fail.* **10**, 1–6 (2017).
19. Sun, H., Davison, B. A., Cotter, G., Pencina, M. J. & Koch, G. G. Evaluating treatment efficacy by multiple end points in phase ii acute heart failure clinical trials analyzing data using a global method. *Circ. Hear. Fail.* **5**, 742–749 (2012).
20. Felker, G. M. & Maisel, A. S. A global rank end point for clinical trials in acute heart failure. *Circ. Heart Fail.* **3**, 643–646 (2010).
21. Margulies, K. B. *et al.* Effects of liraglutide on clinical stability among patients with advanced heart failure and reduced ejection fraction: A randomized clinical trial. *JAMA - J. Am. Med. Assoc.* **316**, 500–508 (2016).
22. Brown, P. M. & Ezekowitz, J. A. Power and sample size estimation for nonparametric composite endpoints: Practical implementation using data simulations. *J. Mod. Appl. Stat. Methods* **16**, 215–230 (2017).
23. Guan, W.-J. *et al.* Clinical Characteristics of Coronavirus Disease 2019 in China. *N. Engl. J. Med.* **19**, 19–20 (2020).
24. Zhou, F. *et al.* Clinical course and risk factors for mortality of adult inpatients with COVID-19 in Wuhan, China: a retrospective cohort study. *Lancet* **395**, 1054–1062 (2020).
25. Sharma, A. *et al.* Use of Biomarkers to Predict Specific Causes of Death in Patients With Atrial Fibrillation. *Circulation* **138**, 1666–1676 (2018).
26. Clerkin, K. J. *et al.* Coronavirus Disease 2019 (COVID-19) and Cardiovascular Disease. *Circulation* **2019**, (2020).
27. Nerenberg, K. A. *et al.* Hypertension Canada’s 2018 Guidelines for Diagnosis, Risk Assessment, Prevention, and Treatment of Hypertension in Adults and Children. *Can. J. Cardiol.* **34**, 506–525 (2018).

26. Appendix

Appendix: CTN CIHR Canadian HIV Trials Network Comments

<u>Reviewer #1:</u>

Proposal Strengths:

- Important question that impacts a large number of individuals.
- Simple intervention.

Proposal Weakness:

- Primary outcome is hard to interpret.
- Sample size is inadequate. Justification would be strengthened if the investigators demonstrated collaboration with other groups to bolster the sample size (even if it would be a meta-analysis, rather than a multi-centre trial) – how does the proposed trial mesh with the French trial referred to in the Justification of Equipoise section?

Response: We recognize that the primary outcome cannot be adequately powered with traditional time to event outcomes. Thereby we are employing a well-used methodology of global rank sum score to enable us to complete this analysis and to power it sufficient to give a global answer of whether our intervention can improve outcomes. We are reaching out to the French and other teams to determine how we can post-hoc combine data. If there is a possibility of conducting this post-hoc combination, we will submit an IRB amendment.

<u>Reviewer #2:</u>

Proposal Strengths:

This is an important question to answer as many of the patients with COVID-19 have CVD comorbidities and are, therefore, likely on either ACEi or ARB. The inclusion, exclusion criteria seem appropriate, with one exception discussed below. The applicants are cognizant of the challenges with other COVID-19 trials going on and have tried to minimize the impact of this study on already overwhelmed staff. The choice and weighting of the individual components of the outcome appear appropriate, though some vetting with healthcare professionals would have strengthened the validity of the components and the scoring

Response: The weights we have ascribed are what have been used in prior analyses (as we have referenced) and justify their use in the current trial.

Proposal Weaknesses:

Comment: Why non-inferiority? The definitive trial will not be set up as a non-inferiority trial given there is no interest in demonstrating that stopping RAAS inhibitors is non-inferior to continuing to use them. The research approach should seek to clarify whether withholding RAAS inhibitors leads to better outcomes when compared to continuing them.

Response: We are hypothesizing that continuing ACEi/ARB may be beneficial compared to stopping these therapies. We have clarified this in the protocol. We discuss the statistical approach below.

Comment: It also isn't clear why the global score, which is ordinal, is summarized using means and SDs? There is also no justification for a choice of a 4-unit difference as the boundary for non-inferiority? While the applicants stress the fact that ACEi/ARB upregulates the ACE2 receptor, they fail to address the premise that ACEi/ARB use may actually be beneficial, given that COVID-19 down regulates ACE2 which can increase the severity of lung injury. These counter arguments could be better presented, especially as they speak directly to the controversy. While this reference was only just published and would not have been

available to the applicants, it would help strengthen the background section by providing both aspects of ACE2. https://doi.org/10.1161/JAHA.120.016219

The reference to support the use of a global score is not ideal. A better reference, which also includes information on how to power a study using a global score would be Brown PM et al. J Modern Applied Stat Methods 2017;16(2)215-230.
Whether the global score is hierarchical or, as stated in several places, based on whichever event occurs first, is confusing. Adding to the confusion is the statement that an earlier death is ranked 'first then later deaths.' What does this mean and how is the ranking changed based on the time of death?

Response: We have added this reference and modified our endpoint to occur from baseline to 7 days. We have removed reference to hierarchy. We have made the following changes to our analysis based on your suggestions:

"Patients who died during the 7th day of the study will be ranked based on all events occurring before their death and also including the fatal event in the score. Next, patients who did not die but were transferred to ICU for invasive ventilation will be ranked based on all the events occurring before the ICU entry and also including the ICU admission in the score. Those patients who neither died nor were transferred to ICU for invasive ventilation will be ranked based on the subsequent outcomes (and so on for all of the outcomes). The mean rank score will then be compared between groups. In this scheme, a lower mean rank score indicates greater overall stability for participants..“

Comment: The study runs to day 7 or date of discharge, so why the focus on the global score at day 4. Is there data to suggest that most COVID-19 patients are already discharged at 4 days with not enough patients still in hospital up to day 7? Provide a rationale for this choice of 4 days for the primary endpoint.

Response: We have modified the study for the primary endpoint to be assessed from baseline to 7 days to maximize the possible differences in outcomes we will ascertain.

Comment: There is no description of the statistical analyses that will be undertaken. From the sample size calculation, I can infer how the primary endpoint will be assessed, but there is no indication how the individual components will be compared between the groups for the secondary endpoints.

Response: Our statistical analysis plan is documented in 'Primary endpoint'. We have created a new section entitled 'Statistical analysis plan' and we have added the following:
"Medians, 25th and 75th percentiles will be presented for continuous variables; the number and percentage of patients in each category will be presented for categorical variables. For all endpoints a p-value ≤0.05 will be considered statistically significant. Appropriate statistical models will be used to examine the effect of the withdrawl intervention on both the primary, secondary, and exploratory outcomes. For the global rank-based endpoints, a non-parametric testing strategy will be employed. For continuous endpoint variables, conventional general linear models will be used. For endpoints where the response is dichotomous (binary), the logistic regression model will be used."

Comment: I completely understand that signed consent is not practical in the COVID-19 setting, but it would be important to follow-up on verbal consent with a signature at discharge.

Response: There may be many patients who will be discharged as they are clinically stable but will still be contagious. As a result, for the safety of study staff we will not require written signature.

Comment: Initially the exclusions include uncontrolled HTN with the use of 5 BP meds. However, in the text it is stated that anyone with 5 or more blood pressure meds will be excluded. The latter makes more sense and this should be consistent in the protocol.

Response: We have modified to keep the protocol consistent with the latter.

Comment: Hs-cTn is not in the CRF, even though it is an endpoint. Please support the choice of 30% increase without any reference to 95th percentile cut-point in the evaluation of hs-cTn. The increase in CRP references 48 hours, but it looks like blood is only collected at baseline and 4 days. Please reconcile.

Response: A 30% increase in troponin is a reasonable threshold for a binary increase as the risk of outcomes in other cardiovascular studies significantly increases at this threshold. We have reconciled the 48 hours of CRP to indicate that the primary outcome will be measured at baseline and day 7/discharge. We will also be assessing biomarker at day 4.

Comment: Not clear how you can report on biomarker trends, as per the comment in the 'Implications' section, when for most patients you will have only two measurements – baseline and 4 days.

Response: We have changed the protocol to have the primary endpoint from baseline to day 7/discharge with additional biomarkers measured at day 4. This will give us the ability to evaluate a trajectory over multiple measurements.

Comment: Appendix Table 1 – ACEi are reported as trade names with generic names in brackets; ARBS are reported as generic with trade names in brackets. Would suggest the latter presentation and consistency across both classes of meds.

Response: We have made these changes.

Comment: CRF – in addition to missing hs-cTn measurements and no mention of CRP at 48 hours, many of the clinical events are missing. Please revise. Also note, here again reference is made to 'first event'. This makes no sense, as you would score new onset AF if it occurred first and ignore death if it came after the AF.

Response: We have modified the CRF to capture all possible endpoints and we have removed the reference to first event.

<u>Reviewer #3:</u>

Proposal Strengths:

Comments: This is a timely and important question that has equipoise and potentially significant implications for adult patients with hypertension or other cardiovascular diseases who are admitted with COVID-19.

Proposal Weaknesses:

Comment: Given that the rationale for the study is, at least in part, related to the hypothesis that ACE-2 is up-regulated by ACEi and ARBs, it is not at all clear that study duration is sufficient (4 days). In other words, how likely is it that the biological effect of ACEi and ARBs on the renin-angiotensin-aldosterone system would be significantly altered over the 4 day study period? Some discussion of this is needed in the background. If, on the other hand, the hypothesis is that no effect is expected over this short study duration, this should be included as part of the explanation in the background or rationale.

Response: We have now changed the primary endpoint to be assessed from baseline to day 7 (or discharge) to maximize the possibility of identifying a possible benefit between the intervention groups.

Comment: No information is provided on how long SARS-CoV-2 test results are expected to take in the study sites. How will the delay in obtaining test results impact time of recruitment and therefore the likelihood of finding a useful study result?

Response: At our institutions these are done within 4-8 hours, thereby enabling timely access to test results.

Comment: There is no description of the research team, though 3 investigators are listed on the consent form. There does not appear to be any Infectious Diseases involvement.

Response: We had not added the full research team to the ICF or protocol. We have an infectious disease expert (Dr. Nadine Kronfi) who will be contributing as a co-investigator on this project.

4. The sample size seems small for a non-inferiority trial. A more detailed statistical explanation would be helpful.

Response: We have modified our protocol to the following:

"All analyses will be conducted using the intention-to-treat principle and will include all randomized patients. Analysis of the global rank end point was based on the Wilcoxon test statistic. Medians, 25th and 75th percentiles will be presented for continuous variables; the number and percentage of patients in each category will be presented for categorical variables. For all endpoints a p-value ≤0.05 will be considered statistically significant. Appropriate statistical models will be used to examine the effect of the withdrawal intervention on both the primary, secondary, and exploratory outcomes. For the global rank-based endpoints, a non-parametric testing strategy will be employed. For continuous endpoint variables, conventional general linear models will be used. For endpoints where the response is dichotomous (binary), the logistic regression model will be used."

Appendix Table 1: List of currently approved angiotensin converting enzyme inhibitors (ACEi) and angiotensin receptor blockers (ARB) approved in Canada

ACEi	ARB
Captopril<br>Enalapril<br>Lisinopril<br>Benazepril<br>Fosinopril	Candesartan<br>Eprosartan<br>Irbesartan<br>Telmisartan<br>Valsartan

Ramipril<br>Quinapril<br>Perindopril<br>Trandolapril<br>Moexipril	Losartan<br>Olmesartan

Appendix Table 2: List of alternative therapies for treating team to consider with temporary cessation of angiotensin converting enzyme inhibitors (ACEi) and angiotensin receptor blockers (ARB). Adapted from Canadian Hypertension Guidelines[27]

Indication for ACEi or ARB	Alternative classes	Examples of medications, starting dosing and range
Systolic or diastolic hypertension	1) Thiazide/thiazide-like diuretics,<br>2) Long acting non-dihydropyridine calcium channel blockers	Thiazide/Thiazide like-diuretic:<br>Hydrochlorothiazide 25mg per day (range 25-100 mg)<br>Indapamide 1.25 mg per day(1.25-5mg)<br>Non-dihydropyridine calcium channel blockers<br>Nifedipine XL 30 mg daily (30-60 mg daily)<br>Felodipine ER 5 mg daily (5-10 mg daily)<br><br>Amlodipine 5mg daily (5-10 mg daily)
Coronary artery disease, diabetes without hypertension, or chronic kidney disease with proteinurea	Long-acting calcium channel blockers	Nifedipine XL 30 mg daily (30-60 mg daily)<br>Felodipine ER 5 mg daily (5-10 mg daily)<br><br>Amlodipine 5mg daily (5-10 mg daily)

Appendix Table 3: Case report form

**Demographic and admission history data**
Date of admission:
Date of COVID diagnosis:
**Baseline demographics**
Age (years) at admission
Sex at birth – male vs female
Race<br>O American Indian, First Nations, or Aboriginal<br>O Black or African American<br>O White or Caucasian<br>O Not Reported or Refused
**Vital signs at baseline**
Height _________ O cm O in Weight _________ O kg O lb<br>Resting Heart Rate: ___________ (beats/min)<br>Resting Systolic Blood Pressure: ___________ (mmHg)<br>Resting Diastolic Blood Pressure: __________ (mmHg)

Resting Respiratory Rate: _______ (respirations/min)
**<u>Laboratory findings to be captured at randomization, day 4 and day 7 or day of discharge</u>**
**Randomization**<br>Serum Hemoglobin ____ O g/dL O mmol/L<br>Serum Sodium ____ O mEq/L O mmol/L<br>Serum Potassium ____ O mEq/L O mmol/L<br>Blood Urea Nitrogen ____ O mg/dL O mmol/L<br>Serum Creatinine ____ O mg/dL O mmol/L<br>Natriuretic Peptides ____ O BNP O NT-proBNP (pg/mL)<br>Serum Glucose ____ O mEq/L O mmol/L<br>C-reactive protein: ____ O mEq/L O mmol/L<br>Lymphocyte count: ____<br>High-sensitivity Troponin T:_____ O mEq/L O mmol/L<br><br>**Day 4**<br>Serum Hemoglobin ____ O g/dL O mmol/L<br>Serum Sodium ____ O mEq/L O mmol/L<br>Serum Potassium ____ O mEq/L O mmol/L<br>Blood Urea Nitrogen ____ O mg/dL O mmol/L<br>Serum Creatinine ____ O mg/dL O mmol/L<br>Natriuretic Peptides ____ O BNP O NT-proBNP (pg/mL)<br>Serum Glucose ____ O mEq/L O mmol/L<br>C-reactive protein: ____ O mEq/L O mmol/L<br>Lymphocyte count: ____<br>High-sensitivity Troponin T:_____ O mEq/L O mmol/L<br><br>**Day 7 or day of discharge**<br>Serum Hemoglobin ____ O g/dL O mmol/L<br>Serum Sodium ____ O mEq/L O mmol/L<br>Serum Potassium ____ O mEq/L O mmol/L<br>Blood Urea Nitrogen ____ O mg/dL O mmol/L<br>Serum Creatinine ____ O mg/dL O mmol/L<br>Natriuretic Peptides ____ O BNP O NT-proBNP (pg/mL)<br>Serum Glucose ____ O mEq/L O mmol/L<br>C-reactive protein: ____ O mEq/L O mmol/L<br>Lymphocyte count: ____<br>High-sensitivity Troponin T:_____ O mEq/L O mmol/L<br><br>**Day 30 day of discharge**<br>Is the patient alive?: Yes or No - Date<br>Was the patient readmitted?: Yes or No - Date<br>If Yes Readmitted – Date the patient was discharged
**<u>Past medical history (all yes/no unless otherwise stated)</u>**
Prior history of heart failure - O yes O no
Coronary artery disease or prior myocardial infarction - O yes O no
History of Hypertension - O yes O no<br>History of Atrial fibrillation/flutter - O yes O no<br>History of stroke or transient ischemic attack - O yes O no<br>History of Diabetes Mellitus - O yes O no

History of Smoking - O yes O no<br>History of chronic obstructive pulmonary disease - O yes O no<br>History of Sleep Apnea - O yes O no<br>History of Depression - O yes O no<br>History of Dyslipidemia - O yes O no<br>History of Cancer Requiring Chemotherapy or Radiation - O yes O no
History of Chronic liver disease- O yes O no
History of Chronic kidney disease - O yes O no
**Medication at baseline**
Renin-Angiotensin System Inhibitors (total daily dose)<br>O Benazepril ____ mg<br>O Captopril ____ mg<br>O Enalapril ____ mg<br>O Fosinopril ____ mg<br>O Lisinopril ____ mg<br>O Moexipril ____ mg<br>O Perindopril ____ mg<br>O Quinapril ____ mg<br>O Ramipril ____ mg<br>O Trandolapril ____ mg<br>O Azilsartan ____ mg<br>O Candesartan ____ mg<br>O Eprosartan ____ mg<br>O Irbesartan ____ mg<br>O Losartan ____ mg<br>O Olmesartan ____ mg<br>O Telmisartan ____ mg<br>O Valsartan ____ mg
Beta-Adrenergic Receptor Blockers O yes O no (could include any of the following)<br>O Acebutolol<br>O Atenolol<br>O Betaxolol<br>O Bisoprolol<br>O Bucindolol<br>O Carvedilol<br>O Labetalol<br>O Metoprolol tartrate<br>O Metoprolol succinate<br>O Nadolol<br>O Nebivolol<br>O Penbutolol<br>O Pindolol<br>O Propranolol
Mineralocorticoid Receptor Antagonists<br>Spironolactone O yes O no<br>Eplerenone O yes O no
Any Calcium Channel Antagonist O yes O no<br>Aspirin O yes O no<br>Warfarin O yes O no<br>Any Direct Oral Anti-Coagulant O yes O no<br>Any Statin O yes O no<br>Any Additional Anti-Hypertensive Medication O yes O no<br>Chronic NSAID O yes O no

**Antivirals used for treatment of COVID-19**
Hydroxychloroqine O yes O no
Azithromyacin O yes O no
Lopinavir O yes O no
Ritonavir O yes O no
Other:
**Clinical events**
All-Cause Mortality O yes O no ________ (date)<br>Transfer to ICU primarily for Invasive ventilation O yes O no ________ (date)<br>Transfer to ICU for other indication O yes O no ________ (date)<br>Myocardial infarction O yes O no ________ (date)<br>Stroke O yes O no ________ (date)<br>Acute heart failure O yes O no ________ (date)<br>New onset atrial fibrillation O yes O no ________ (date)<br>Length of stay > 4 days O yes O no ________ (date)<br>Day of discharge O yes O no ________ (date)<br>Urgent intravenous treatment for high blood pressure/hypertensive crisis O yes O no ________ (date)<br>Date of RAAS inhibitor re-initiation: ______ (date)<br>What RAAS inhibitor prescribed on discharge: O yes O no
